# A risk model for the early diagnosis of acute myocardial infarction in patients with chronic kidney disease

**DOI:** 10.3389/fcvm.2023.1253619

**Published:** 2023-10-10

**Authors:** Xiao-Feng Su, Xu Chen, Tao Zhang, Jun-Mei Song, Xin Liu, Xing-Li Xu, Na Fan

**Affiliations:** ^1^ Ultrasound in Cardiac Electrophysiology and Biomechanics Key Laboratory of Sichuan Province, Sichuan Provincial People's Hospital, University of Electronic Science and Technology of China, Chengdu, China; ^2^ Department of Cardiovascular Ultrasound & Noninvasive Cardiology, Sichuan Provincial People's Hospital, University of Electronic Science and Technology of China, Chengdu, China

**Keywords:** acute myocardial infarction, chronic kidney disease, early diagnose, SPPH-AMI-model, test accuracy

## Abstract

**Introduction:**

Acute myocardial infarction (AMI) remains a critical disease, characterized by a high fatality rate in several countries. In clinical practice, the incidence of AMI is increased in patients with chronic kidney disease (CKD). However, the early diagnosis of AMI in the above group of patients is still poor.

**Methods:**

In the present study, a total of 829 patients with CKD, defined by an estimated glomerular filtration rate (eGFR) of <60 ml/min/1.73 m^2^ or 60–90 ml/min/1.73 m^2^ for patients with mildly reduced kidney function, who attended the Sichuan Provincial People's Hospital (SPPH) between January 2018 and November 2022 were enrolled. All patients underwent coronary angiography due to the presence of typical or atypical symptoms of AMI. Patients were divided into the following two groups: The training cohort, including 255 participants with AMI and 242 without AMI; and the testing cohort, including 165 and 167 subjects with and without AMI, respectively. Furthermore, a forward stepwise regression model and a multivariable logistic regression model, named SPPH-AMI-model, were constructed to select significant predictors and assist the diagnosis of AMI in patients with CKD, respectively.

**Results:**

The following factors were evaluated in the model: Smoking status, high sensitivity cardiac troponin I, serum creatinine and uric acid levels, history of percutaneous coronary intervention and electrocardiogram. Additionally, the area under the curve (AUC) of the receiver operating characteristic curve were determined in the risk model in the training set [AUC, 0.78; 95% confidence interval (CI), 0.74–0.82] vs. the testing set (AUC, 0.74; 95% CI, 0.69–0.79) vs. the combined set (AUC, 0.76; 95% CI, 0.73–0.80). Finally, the sensitivity and specificity rates were 71.12 and 71.21%, respectively, the percentage of cases correctly classified was 71.14%, while positive and negative predictive values of 71.63 and 70.70%, respectively, were also recorded.

**Discussion:**

The results of the current study suggested that the SPPH-AMI-model could be currently considered as the only risk scoring system for the early diagnosis of AMI in patients with CKD. This method could help clinicians and emergency physicians to quickly and accurately diagnose AMI in patients with CKD to promote the immediate and effective treatment of these patients.

## 1. Introduction

Acute myocardial infarction (AMI) is characterized by myocardial cell death caused by prolonged myocardial ischemia and hypoxia. AMI is considered as a sever disease since it is characterized by a high fatality rate. Delayed diagnosis of AMI could prevent the immediate treatment of patients with effective therapies (1). Therefore, the early diagnosis of AMI is crucial for its treatment. The diagnosis of AMI in patients with chronic kidney disease (CKD) needs more attention. This finding could be due to the fact that several patients with CKD do not experience the classic clinical symptoms of AMI (2, 3). Secondly, several electrocardiography (ECG) changes, such as ST deviations and T-wave inversion, could occur due to left ventricular hypertrophy. The above changes could mimic or obscure AMI (4). Thirdly, cardiac troponin (cTn) levels and more particularly those of high-sensitivity cTn (hs-cTn) are often elevated in patients with CKD, thus reducing their diagnostic effectiveness. Several previous studies also suggested that the assessment of hs-cTn levels could display a lower clinical specificity for AMI in the setting of CKD (5–9). Additionally, it has been reported that patients with CKD are more likely to experience adverse events associated with coronary intervention (10). Therefore, the early diagnosis of AMI in patients with CKD remains a challenge for clinicians. According to the 2021 ACC/AHA guidelines, clinicians should be aware that in elderly patients with renal disease the assessment of changes in serial measurements is very significant for improving diagnostic specificity (11). However, currently, no studies have been conducted on the development of a risk scoring system for predicting AMI in patients with CKD via analyzing several risk factors, such as arterial hypertension, dyslipidaemia and diabetes mellitus (DM) (12, 13). Therefore, the current study aimed to evaluate all associated risk factors and indicators to establish a scoring model for the early diagnosis of AMI in patients with CKD.

## 2. Methods

### 2.1. Patient selection

In the present study, patients who experienced the typical or atypical symptoms of myocardial ischemia, including chest pain, chest distress, dyspnea, palpitations or fatigue, and diagnosed with CKD [estimated glomerular filtration rate (eGFR), < 60 ml/min/1.73 m^2^], mildly reduced kidney function (eGFR, 60–90 mL/min/1.73 m^2^) or other CKD-related diseases, such as chronic glomerulonephritis (13) or albuminuria (2) were enrolled. AMI was diagnosed, according to the universal definition of AMI (14), based on the patient's medical history, laboratory tests, including hs-cTnI levels, electrocardiography, echocardiography and coronary angiographic morphology assessment. Therefore, a total of 1,504 patients with CKD who underwent coronary angiography, due to the onset of typical or atypical symptoms of AMI, at the Sichuan Provincial People's Hospital (SPPH) between January 2018 and November 2022 were included in the study. Additionally, both 12-lead ECG and laboratory tests, such as hs-cTnI, were performed within 24 h after the onset of the symptoms. Patients (*n* = 569) with mildly reduced kidney function (eGFR, 60–90 ml/min/1.73 m^2^), but without CKD, were excluded from the study. In addition, patients with missing data (*n* = 106) were also excluded. Finally, the data of a total of 829 participants, including 420 patients with AMI and 409 without AMI, were analyzed.

### 2.2. Data acquisition

Several risk factors have been identified in previous studies to be associated with AMI. Therefore, in the present study all these factors, including age, sex, smoking status, obesity, family history of coronary artery disease (CAD), arterial hypertension and DM, atrial fibrillation, peripheral vascular disease, history of valvular heart diseases (*n* = 68) or cardiomyopathies, such as dilated cardiomyopathy (*n* = 7) and hypertrophic cardiomyopathy (*n* = 3), history of cerebral infarction and history of percutaneous coronary intervention (PCI), were evaluated. Relevant laboratory tests, such as the assessment of blood lipid, myocardial enzyme, eGFR, serum creatinine (Scr) and uric acid (UA) levels, and ECG were also performed. ECG results were evaluated independently by a diagnostician blinded to the other data. Changes in the ECG results were considered positive when ST deviations of ±1 mm in two contiguous leads (II, III and avF or I, avL, V5, V6 or V1–V4), ST deviations of ±1 mm in avR or V1 lead and hyperacute T wave or T-wave inversion as coronal T-wave were recorded. All the other ECG findings were considered negative. All the aforementioned factors are listed in Table 1.

**Table 1:** The comparison of factors between AMI group and non-AMI group in the training,testing and combined set.

Variables	–	Training set				Testing set				Combined set			
		Non-AMI (*N* = 242)	AMI (*N* = 255)	Statistic	*P*	Non-AMI (*N* = 167)	AMI (*N* = 165)	Statistic	*P*	Non-AMI (*N* = 409)	AMI (*N* = 420)	Statistic	*P*
Sex	Female	81 (33.47)	64 (25.1)	*χ*^2 ^= 3.82	0.051	52 (31.14)	47 (28.48)	*χ*^2 ^= 0.17	0.683	133 (32.52)	111 (26.43)	*χ*^2 ^= 3.41	0.065
	Male	161 (66.53)	191 (74.9)			115 (68.86)	118 (71.52)			276 (67.48)	309 (73.57)		
Age	Median (Q1,Q3)	68.50 (62.00, 75.00)	70.00 (60.50, 75.00)	*H *= 0.06	0.803	67.00 (59.50, 75.50)	69.00 (63.00, 75.00)	*H *= 3.43	0.064	68.00 (61.00, 75.00)	69.00 (61.00, 75.00)	*H *= 1.88	0.170
Obesity	0	230 (95.04)	241 (94.51)	*χ*^2 ^*<* 0.01	0.949	156 (93.41)	150 (90.91)	*χ*^2 ^*=* 0.42	0.519	386 (94.38)	391 (93.1)	*χ*^2^ = 0.38	0.537
	1	12 (4.96)	14 (5.49)			11 (6.59)	15 (9.09)			23 (5.62)	29 (6.9)		
Diabetes mellitus	0	155 (64.05)	136 (53.33)	*χ*^2 ^= 5.44	0.020	115 (68.86)	79 (47.88)	*χ*^2 ^= 14.75	<0.01	270 (66.01)	215 (51.19)	*χ*^2 ^= 18.54	<0.001
	1	87 (35.95)	119 (46.67)			52 (31.14)	86 (52.12)			139 (33.98)	205 (48.81)		
Hypertension	0	62 (25.62)	53 (20.78)	*χ*^2 ^= 1.37	0.241	43 (25.75)	33 (20)	*χ*^2^* *= 1.25	0.264	105 (25.67)	86 (20.48)	*χ*^2 ^= 2.87	0.090
	1	180 (74.38)	202 (79.22)			124 (74.25)	132 (80)			304 (74.33)	334 (79.52)		
Drinking	0	184 (76.03)	189 (74.12)	*χ*^2 ^= 0.15	0.697	128 (76.65)	113 (68.48)	*χ*^2 ^= 2.38	0.123	312 (76.28)	302 (71.90)	*χ*^2 ^= 1.85	0.174
	1	58 (23.97)	66 (25.88)			39 (23.35)	52 (31.52)			97 (23.72)	118 (28.10)		
Smoking	0	161 (66.53)	143 (56.08)	*χ*^2 ^= 5.28	0.022	98 (58.68)	92 (55.76)	*χ*^2^ = 0.18	0.669	259 (63.33)	235 (55.95)	*χ*^2^ = 4.38	0.036
	1	81 (33.47)	112 (43.92)			69 (41.32)	73 (44.24)			150 (36.67)	185 (44.05)		
Family history	0	238 (98.35)	241 (94.51)	*χ*^2^ = 4.20	0.041	164 (98.2)	161 (97.58)	*Fisher*	0.722	402 (98.29)	402 (95.71)	*χ*^2^ = 3.86	0.050
	1	4 (1.65)	14 (5.49)			3 (1.8)	4 (2.42)			7 (1.71)	18 (4.29)		
History of cerebral infarction	0	213 (88.02)	213 (83.53)	*χ*^2 ^= 1.69	0.193	140 (83.83)	144 (87.27)	*χ*^2^ = 0.54	0.462	353 (86.31)	357 (85.00)	*χ*^2^ = 0.19	0.661
	1	29 (11.98)	42 (16.47)			27 (16.17)	21 (12.73)			56 (13.69)	63 (15.00)		
Atrial fibrillation	0	208 (85.95)	216 (84.71)	*χ*^2 ^= 0.07	0.791	127 (76.05)	139 (84.24)	*χ*^2^ = 3.00	0.083	335 (81.91)	355 (84.52)	*χ*^2^ = 0.84	0.360
	1	34 (14.05)	39 (15.29)			40 (23.95)	26 (15.76)			74 (18.09)	65 (15.48)		
PDA	0	88 (36.36)	92 (36.08)	*χ*^2 ^< 0.01	>0.999	61 (36.53)	58 (35.15)	*χ*^2^ = 0.02	0.883	149 (36.43)	150 (35.71)	*χ*^2^ = 0.02	0.887
	1	154 (63.64)	163 (63.92)			106 (63.47)	107 (64.85)			260 (63.57)	270 (64.29)		
Cholesterol	Median (Q1, Q3)	3.84 (3.21, 4.75)	3.81 (3.22, 4.64)	*H* = 0.38	0.540	3.92 (3.28, 4.61)	4.03 (3.17, 4.61)	*H *= 0.05	0.823	3.89 (3.22, 4.72)	3.86 (3.20, 4.63)	*H* = 0.36	0.550
TG	Median (Q1, Q3)	1.46 (1.07, 2.1)	1.53 (1.18, 2.2)	*H* = 1.38	0.240	1.51 (1.06, 2.25)	1.54 (1.11, 2.2)	*H* = 0.49	0.485	1.50 (1.07, 2.13)	1.54 (1.15, 2.20)	*H* = 1.75	0.185
LDL	Median (Q1, Q3)	1.92 (1.44, 2.53)	2.04 (1.52, 2.7)	*H* = 1.13	0.288	2.00 (1.57, 2.65)	2.18 (1.54, 2.72)	*H* = 0.52	0.471	1.95 (1.47, 2.57)	2.10 (1.53, 2.72)	*H* = 1.51	0.219
HDL	Median (Q1, Q3)	1.12 (0.9, 1.38)	1.04 (0.88, 1.31)	*H* = 3.82	0.051	1.11 (0.94, 1.38)	1.05 (0.87, 1.23)	*H* = 5.63	0.018	1.12 (0.92, 1.38)	1.04 (0.87, 1.27)	*H* = 9.16	0.002
BNP	Median (Q1, Q3)	120 (62, 255)	141 (64, 390.5)	*H* = 2.03	0.154	130.00 (66.50, 347.00)	168.00 (66.00, 456.00)	*H* = 1.33	0.249	124.00 (65.00, 285.00)	153.00 (64.00, 427.50)	*H* = 3.33	0.068
Lipoprotein A	Median (Q1, Q3)	20.5 (16.22, 26.67)	20.8 (15.9, 26.7)	*H* = 0.13	0.718	20.50 (16.10, 27.05)	20.50 (15.20, 25.00)	*H* = 1.14	0.285	20.50 (16.20, 26.80)	20.60 (15.80, 25.92)	*H* = 0.16	0.689
CK-MB	Median (Q1, Q3)	1.20 (0.80, 2.10)	1.80 (1.00, 5.10)	*H* = 26.46	<0.001	1.10 (0.80, 1.90)	2.00 (1.10, 5.70)	*H* = 31.62	<0.001	1.20 (0.80, 2.00)	1.90 (1.00, 5.40)	*H* = 56.61	<0.001
Myohemoglobin	Median (Q1, Q3)	88.8 (58.95, 186.12)	150.2 (79.25, 368.2)	*H* = 35.27	<0.001	95.70 (63.40, 154.90)	135.10 (71.30, 322.50)	*H* = 12.64	<0.001	93.2 (60.80, 178.00)	146.70 (75.90, 350.95)	*H* = 47.34	<0.001
hs-cTn I	Median (Q1, Q3)	18.60 (6.55, 46.77)	120.20 (18.65, 3,198.20)	*H* = 76.11	<0.001	16.40 (5.45, 43.40)	181.30 (19.00, 1,868.70)	*H* = 58.18	<0.001	17.70 (6.20, 44.90)	142.26 (18.92, 2,988.20)	*H *= 134.05	<0.001
eGFR	Median (Q1, Q3)	37.02 (18.7, 46.73)	27.51 (9.57, 41.27)	*H* = 15.28	<0.001	36.53 (20.14, 47.06)	34.43 (14.09, 45.61)	*H* = 0.19	0.662	36.97 (19.07, 47.00)	30.41 (11.15, 43.61)	*H* = 11.07	<0.001
Scr	Median (Q1, Q3)	152.70 (125.95, 250.07)	192.30 (140.70, 501.02)	*H* = 17.28	<0.001	151.40 (125.70, 259.65)	158.10 (123.70, 341.80)	*H* = 0.02	0.885	151.4 (125.80, 254.30)	180.00 (132.30, 390.30)	*H* = 11.02	<0.001
UC	Median (Q1, Q3)	427.00 (345.00, 519.00)	448.00 (356.00, 550.00)	*H* = 2.14	0.143	442.00 (375.50, 544.00)	455.00 (373.00, 544.00)	*H* = 0.01	0.909	433.00 (357, 528)	450.50 (357.75, 547.25)	*H* = 1.01	0.315
Valvulopathy or cardiomyopathy	0	215 (88.84)	234 (91.76)	*χ*^2 ^= 0.90	0.342	146 (87.43)	156 (94.55)	*χ*^2 ^= 4.29	0.038	361 (88.26)	390 (92.86)	*χ*^2 ^= 4.60	0.032
	1	27 (11.16)	21 (8.24)			21 (12.57)	9 (5.45)			48 (11.74)	30 (7.14)		
History of PCI	0	173 (71.49)	157 (61.57)	*χ*^2^ = 5.04	0.025	115 (68.86)	99 (60)	*χ*^2^ = 2.47	0.116	288 (70.42)	256 (60.95)	*χ*^2^ = 7.81	0.005
	1	69 (28.51)	98 (38.43)			52 (31.14)	66 (40)			121 (29.58)	164 (39.05)		
ECG	0	124 (51.24)	50 (19.61)	*χ*^2^ = 53.22	<0.001	105 (62.87)	40 (24.24)	*χ*^2^ = 48.79	<0.001	229 (55.99)	90 (21.43)	*χ*^2 ^= 103.10	<0.001
	1	118 (48.76)	205 (80.39)			62 (37.13)	125 (75.76)			180 (44.01)	330 (78.57)		

### 2.3. Statistical analysis

All baseline characteristics were described and compared between the AMI and non-AMI groups in the training, testing and combined set. The normally distributed variables are expressed as the mean ± standard deviation (SD). The differences between two groups were compared using t test. Additionally, the non-normally distributed variables are expressed as the median and interquartile range (IQR). The above date was compared using Kruskal–Wallis rank-sum test. The binomial variables are expressed as frequency and proportion, and were compared by Chi-square test or Fisher's exact test. In the training set, a forward stepwise regression model was constructed to select significant predictors and a multivariable logistic regression model was then established. All *p*-values were two-sided and the 95% confidence interval (95% CI) were also presented. All analyses were performed using R software (R Foundation for Statistical Computing, Vienna, Austria).

## 3. Results

### 3.1. Study population

All the 1,504 patients with CKD underwent coronary angiography after the onset of the typical or atypical symptoms of AMI, including acute chest pain, palpitation, dyspnea or syncope. In the present study, not only patients with CKD and eGFR valus of <60 ml/min/1.73 m^2^ were included, but also patients with mild CKD-related diseases (eGFR, 60–90 ml/min/1.73 m^2^), such as nephrotic syndrome (1), chronic glomerulonephritis (13) and secondary albuminuria (17). However, 569 patients with mildly reduced kidney function (eGFR, 60–90 ml/min/1.73 m^2^), but without CKD, and 106 patients with missing data were excluded. The remaining 829 patients were randomly divided into the following two groups: The 60% of patients as training cohort, including 255 patients with AMI and 242 without AMI, and the 40% of patients as testing cohort, including 165 and 167 patients with and without AMI, respectively.

### 3.2. Study factors

In the current study, a total of 13 AMI-related clinical risk factors were evaluated, including 12 laboratory testing factors and ECG data. The comparisons of all factors are shown in Table 1. The analysis revealed that the most common cardiovascular risk factors were sex (male in AMI vs. non-AMI, 73.57 vs. 67.48%), smoking status (AMI vs. non-AMI, 44.05 vs. 36.67%), obesity (AMI vs. non-AMI, 6.9 vs. 5.62%), hypertension (AMI vs. non-AMI, 79.52% vs. 74.33%), DM (AMI vs. non-AMI, 48.81 vs. 33.98%), atrial fibrillation (AMI vs. non-AMI, 15.48 vs. 18.09%) and history of PCI (AMI vs. non-AMI, 39.05 vs. 29.58%). The laboratory testing factors included the levels of cholesterol (AMI vs. non-AMI, 3.86 vs. 3.89), triglyceride (AMI vs. non-AMI, 1.54 vs. 1.50), low-density lipoprotein (AMI vs. non-AMI, 2.10 vs. 1.95), creatine kinase MB (AMI vs. non-AMI, 1.9 vs. 1.2), myoglobin (AMI vs. non-AMI, 146.7 vs. 93.2), hs-cTn (AMI vs. non-AMI, 142.26 vs. 17.7), eGFR (AMI vs. non-AMI, 30.41 vs. 36.97), serum creatinine (AMI vs. non-AMI, 180 vs. 151.4) and UA (AMI vs. non-AMI, 450.5 vs. 433). In addition, the ECG positive sign rate in the AMI group was 78.57% compared with 44.01% in the non-AMI group.

Subsequently, a forward stepwise regression model was established to select significant predictors (Table 1) and a multivariable logistic regression model was then developed (Table 2). In the model, the following factors were included: Smoking status, hs-cTnI, Scr, UA, history of PCI and ECG. The risk score of each factor was calculated when the corresponding value of each variable was entered into the following formula: π=(*Y* = 1) = 1/1 + exp(-score), where score = −2.350 + 0.597 × (smoking = 1) + 0.041 × hs-cTnI per 100 + 0.116 × Scr per 100 + 0.139 × UA per 100 + 0.394 × (history of PCI = 1) + 1.079 × (ECG = 1). Subsequently, each score was inserted into the logistic regression model to determine the probability of AMI. The use of the above risk model (SPPH-AMI-model) could promote the early diagnosis of AMI in patients with CKD. Therefore, these patients could be timely treated with the appropriate treatment approach, thus avoiding the delay in patient therapy due to misdiagnosis.

**Table 2:** The result of univariate logistic regression analysis and the SPPH-AMI-model.

Variables	B	*OR*	95% CI		*z*	*P*
Smoking (1 vs. 0)	0.597	1.817	1.202	2.758	2.82	0.005
Hs-cTn I per 100	0.041	1.042	1.024	1.065	4.18	2.86 × 10^−5^
Scr per 100	0.116	1.122	1.046	1.208	3.15	0.002
UC per 100	0.139	1.149	0.997	1.329	1.91	0.057
History of PCI (1 vs. 0)	0.394	1.483	0.964	2.282	1.80	0.073
ECG (1 vs. 0)	1.079	2.94	1.908	4.579	4.84	1.31 × 10^−6^
Constant term	−2.350					

The SPPH-AMI-model: $\pi (Y=1)=\frac{1}{1+\exp(-Score)}$.

*Score* = −2.350 + 0.597 × (smoking = 1) + 0.041 × hs-cTn I per 100 + 0.116 × Scr.

Per 100 + 0.139 × UA per 100 + 0.394 × (history of PCI = 1) + 1.079 × (ECG = 1).

**Figure 1:**
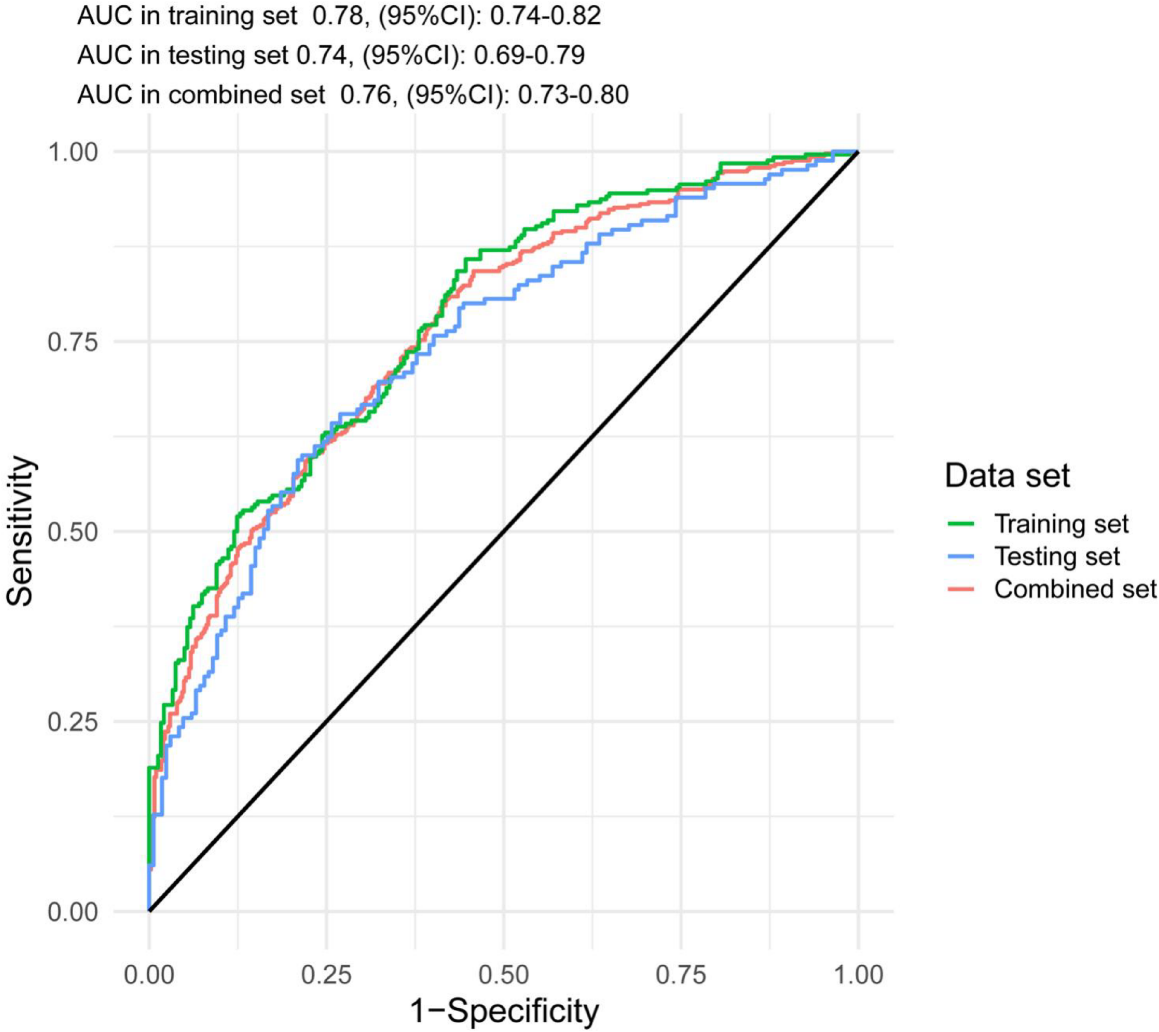
The ROC and AUC of SPHH-AMI-model in training set, testing set and combined set.

In the combined set, the threshold (0.46) of the predicted probability of each case was determined once the balance of sensitivity and specificity was achieved. As shown in Figure 1, the corresponding score was −0.1418. The above finding indicated that when a risk score of >−0.1418 was obtained, patients with CKD could experience AMI.

**Figure 2:**
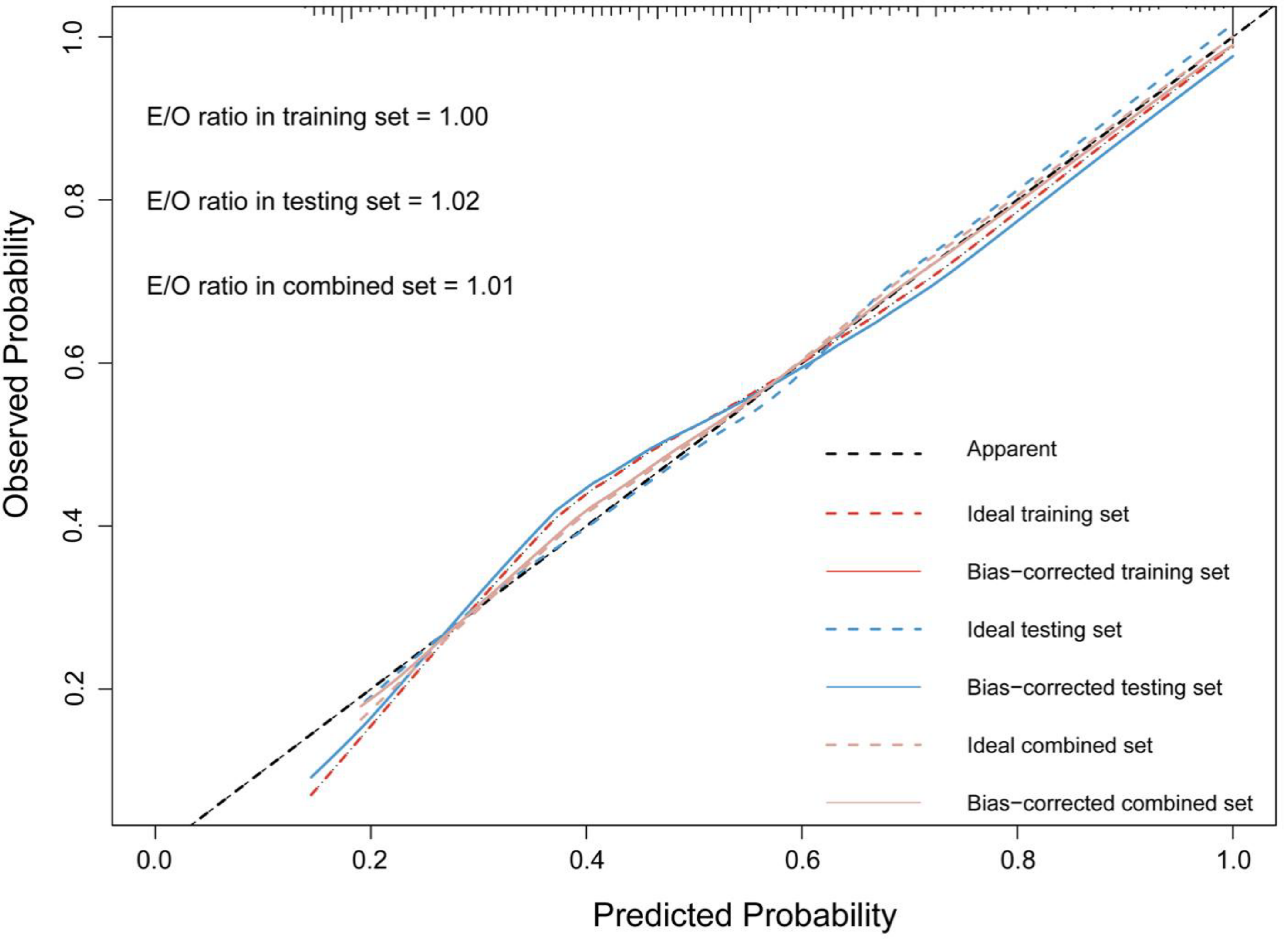
The calibration curve and E:O ratio of SPHH-AMI-model in training set, testing set and combined set.

**Figure 3:**
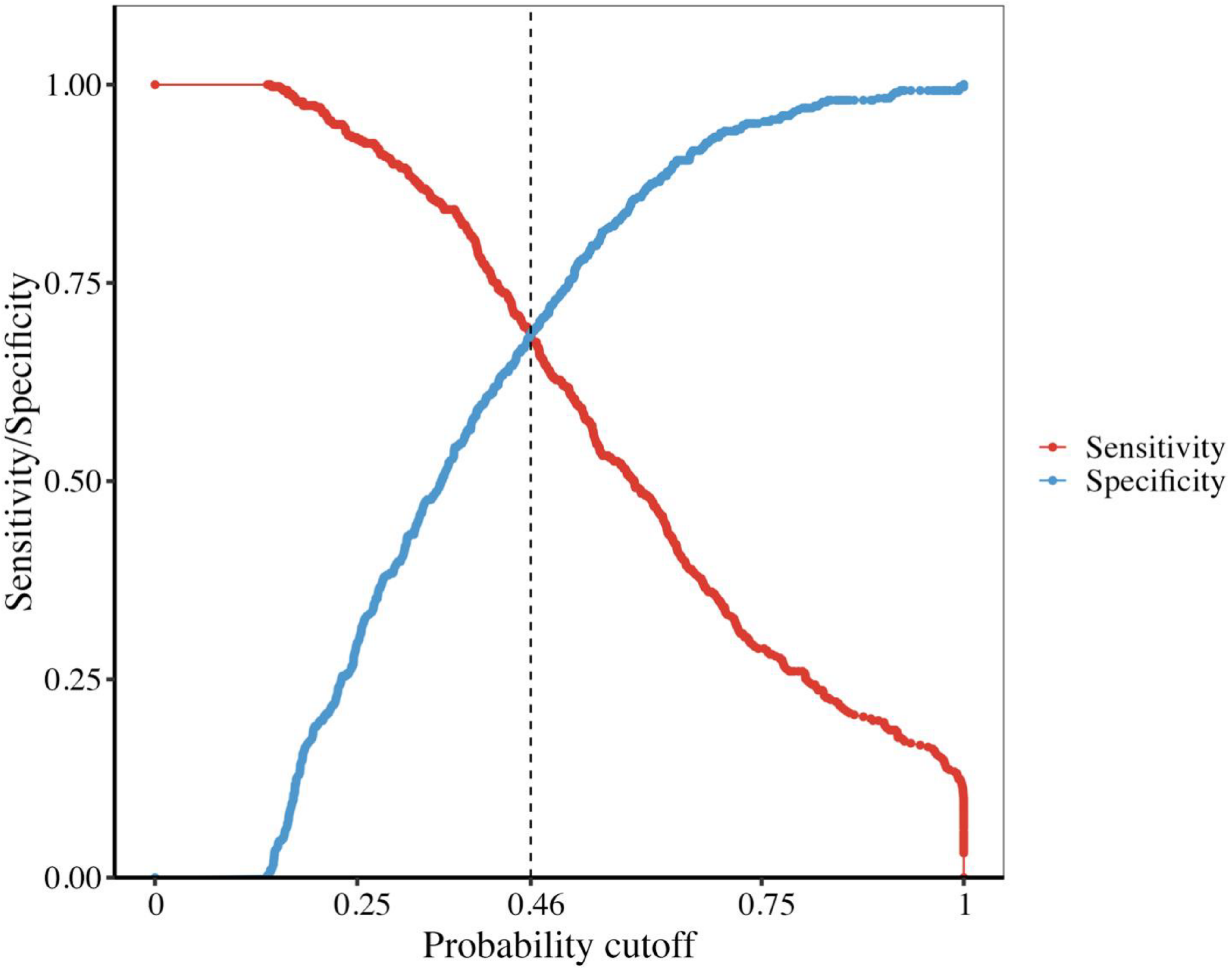
The sensitivity and specificity of our model intersected at the point 0.46 and the corresponding score is −0.1428.

The accuracy of the discrimination of the model was evaluated using receiver operating characteristic (ROC) curve and area under the ROC curve (AUC). As shown in Figure 2, the AUC values of the risk model in the training vs. testing vs. combined sets were 0.78 vs. 0.74 vs. 0.76, respectively. In addition, the model was calibrated using a calibration curve and the observed vs. expected ratio (Figure 3). Furthermore, all parameters in the model were reserved, and the model was independently evaluated in the testing set. In the combined set, the threshold (0.46) of the predicted probability of each case was calculated when the balance of sensitivity and specificity was achieved. As shown in Table 3, the sensitivity and specificity rates were 71.12 and 71.21%, respectively. Additionally, the rate of cases correctly classified was 71.14%, while the positive and negative predictive rates were 71.63 and 70.70%, respectively (Table 3).

**Table 3:** The predictive effectiveness of the model.

Prediction result	True result		合计
	Positive	Negative	
Positive	298	118	416
Negative	121	292	413
合计	419	410	829

Sensitivity: 71.12%.

Specificity: 71.21%.

Positive predictive value (PPV): 71.63%.

Negative predictive value (NPV): 70.70%.

Correction rate: 71.14%.

The association between eGFR and hs-cTnI levels is shown in Table 4. The results demonstrated that the median levels of hs-cTnI increased with the deterioration of renal function in the non-AMI and combined groups.

**Table 4:** The comparison of hs-cTn I in different eGFR groups in training set, testing set and combined set.

Group	eGFR [Median (Q1,Q3)]				*P*
	<15	15–30	30–60	60–90	
Combined group	0.72 (0.27, 7.16)	0.43 (0.15, 12.74)	0.19 (0.06, 1.11)	0.07 (0.03, 1.68)	<0.001
Non-AMI group	0.36 (0.17, 0.71)	0.20 (0.11, 0.42)	0.12 (0.05, 0.38)	0.04 (0.02, 0.15)	<0.001
AMI group	2.23 (0.42, 34.7)	6.28 (0.31, 84.24)	0.47 (0.10, 11.82)	0.43 (0.04, 21.76)	<0.001

## 4. Discussion

Currently, the incidence of AMI- or CAD-related deaths is increasing each year (15). The Fourth Universal Definition of Myocardial Infarction Consensus Document in 2018 provided by the Joint ESC/ACC/AHA/WHF Task Force (14), suggested that the early diagnosis of AMI could depend on the symptoms of myocardial ischemia, the ischemic ECG changes and elevated cTn levels. In fact, diagnosing AMI in patients with CKD could be very difficult. However, previous studies indicated that serial changes on cTn levels could be equally effective in diagnosing AMI in patients with CKD and in those with normal renal function (16, 17). However, the dynamic changes in the levels of cTn could delay the treatment of these patients. The present study aimed to establish a practical and convenient model to promote the early diagnosis of AMI in patients with CKD via comprehensively analyzing relevant clinical risk factors and laboratory test indexes.

Herein, a new scoring system, namely SPPH-AMI-model, which included six novel risk factors, such as smoking status, hs-cTn, Scr and UA levels, history of PCI and ECG, was established. Emerging evidence has suggested that smoking is a major risk factor for CVD (18, 19). This observation is not only due to the fact that smoking has direct toxic effect on myocytes, such as in smoking cardiomyopathy, but also since smoking can cause several comorbidities, such as hypertension and atherosclerotic syndromes, which can also remodel and damage the heart (20). In addition, smoking can also result in vascular stiffness, injury and inflammation, possibly due to the increased levels of several biomarkers (21). It has been reported that impaired kidney function is an independent risk factor for adverse cardiovascular disease outcomes, including AMI, stroke and heart failure (22–25). Other studies also revealed that that higher Scr levels were associated with CVD mortality (26, 27). It has been also previously reported that UA is a significant risk factor for CVD (28). Another study demonstrated that UA could reduce the bioavailability of nitric oxide (NO) via promoting L-arginine degradation, blocking the uptake of L-arginine or scavenging NO from UA-generated oxidants or by UA itself (29). Additionally, UA could induce inflammatory responses (30), which in turn could promote vascular smooth muscle cell proliferation (31). Overall, UA could serve as an intrinsic risk factor in CVD. Interestingly, in the current model, the history of PCI was also a significant risk factor. A previous report on myocardial infarction in Norway showed that a high proportion of patients with AMI had a history of myocardial infarction (32).

Consistent with previous studies (33, 34), the results of the present study also verified that the levels of hs-cTnI were enhanced in several patients with CKD. Several pathological conditions could be involved in the above finding, including anemia, hypotension, small-vessel coronary obstruction, increased ventricular pressure and the direct toxic effects observed in uremic myocardiopathy (35). Overall, the above findings indicated that the increased levels of hs-cTnI could be strongly associated with the diagnosis of AMI in patients with CKD. Therefore, the higher the hs-cTn levels, the stronger the likelihood of developing AMI. Additionally, a previous study suggested that although hs-cTn could exhibit a high diagnostic accuracy in patients with AMI and CKD, the assay-specific optimal cut-off levels of hs-cTn in patients with CKD should be considered higher to ensure the best possible clinical use (4). Therefore, the SPPH-AMI model could more effectively quantify the association between hs-cTnI levels and AMI. In addition, changes in ECG can be also associated with the onset of AMI in clinical practice. Although the challenges in diagnosing AMI in patients with CKD using ECG are great, several patients with AMI and CKD may lack persistent ST-segment elevation. Additionally, it has been reported that ST-segment depression and T-wave inversion are very common in patients with CKD, even in the absence of AMI (36–38). Therefore, the results of the current study suggested that the ECG changes in AMI in patients with CKD, such as ST-segment depression or T-wave inversion, should be considered.

Previous studies also showed that in patients with CKD, regardless the presence of symptoms and clinical risk factors for AMI, ECG and the levels of hs-cTnI exhibited lower-than-expected diagnostic accuracy for AMI (5, 15, 39). Herein, all relevant clinical risk factors and laboratory test indexes, including several new biomarkers, such as B-type natriuretic peptide, were evaluated to establish the SPPH-AMI risk model for the early diagnosis of AMI in patients with CKD. Currently, no similar models have been developed. To the best of our knowledge, the SPPH-AMI-model is currently the only available risk scoring system, which can be used to help clinicians and emergency physicians to directly diagnose AMI in patients with CKD, thus preventing delayed treatment. Furthermore, herein, unlike other studies, patients with CKD-related mild renal insufficiency (eGFR, 60–90 ml/min/1.73 m^2^) were also investigated.

However, the present study has some limitations. Firstly, the current study was a retrospective one. Therefore, further larger multicenter prospective studies are needed to verify the diagnostic value of the SPPH-AMI-model. As shown in Table 3, the correction rate of the model was unsatisfactory. This finding could be due to several reasons. Firstly, this was a retrospective study. Secondly, AMI in patients with CKD could be more insidious and the individual differentiation could be therefore greater. Furthermore, the association of AMI with other significant novel biomarkers, such as procalcitonin and Soluble ST2 (sST2), were not evaluated. Overall, further large multicenter prospective studies are required to identify novel biomarkers or risk factors for establishing a more accurate risk prediction model.

## Data Availability

The data analyzed in this study is subject to the following licenses/restrictions: The original dataset contains some personal information. Requests to access these datasets should be directed to s18583798060@163.com.
